# Protective Role for Properdin in Progression of Experimental Murine Atherosclerosis

**DOI:** 10.1371/journal.pone.0092404

**Published:** 2014-03-25

**Authors:** Tanja Steiner, Lorenza Francescut, Simon Byrne, Timothy Hughes, Archana Jayanthi, Irina Guschina, John Harwood, Katherine Cianflone, Cordula Stover, Sheila Francis

**Affiliations:** 1 Department of Cardiovascular Science, Medical School, University of Sheffield, Sheffield, United Kingdom; 2 Department of Infection, Immunity and Inflammation, University of Leicester, Leicester, United Kingdom; 3 Institute of Infection & Immunity, School of Medicine, Cardiff University, Cardiff, United Kingdom; 4 School of Biosciences, Cardiff University, Cardiff, United Kingdom; 5 Centre de Recherche de l’Institut Universitaire de Cardiologie et de Pneumologie de Québec (CRIUCPQ), Université Laval, Québec, Canada; University Hospital Würzburg, Germany

## Abstract

Genetic, dietary and immune factors contribute to the pathogenesis of atherosclerosis in humans and mice. Complement activation is an integral part of the innate immune defence but also shapes cellular responses and influences directly triglyceride synthesis. Deficiency of Factor B of the alternative pathway (AP) of complement is beneficial in LDLR^−/−^ mice fed a high fat diet. The serum glycoprotein properdin is a key positive regulator of the AP but has not been studied in experimental atherosclerosis. Atherosclerosis was assessed after feeding low fat (LFD) or high fat (HFD) Western type diets to newly generated LDLR^−/−^ Properdin^KO^ (LDLR^−/−^P^KO^) and LDLR^−/−^P^WT^ mice. Lipids, lymphocytes and monocytes were similar among genotypes, genders and diets. Complement C3, but not C3a_desarg_, levels were enhanced in LDLR^−/−^P^KO^ mice regardless of diet type or gender. Non-esterified fatty acids (NEFA) were decreased in male LDLR^−/−^P^KO^ fed a HFD compared with controls. All mice showed significant atherosclerotic burden in aortae and at aortic roots but male LDLR^−/−^ mice fed a LFD were affected to the greatest extent by the absence of properdin. The protective effect of properdin expression was overwhelmed in both genders of LDLR^−/−^mice when fed a HFD. We conclude that properdin plays an unexpectedly beneficial role in the development and progression of early atherosclerotic lesions.

## Introduction

Atherosclerosis is a progressive inflammatory condition resulting in cellular changes in the arterial intima with fatty deposits, so-called atheroma. Oxidatively modified low density lipoproteins (oxLDLs) are involved and are associated with elevated levels of lipoproteins in blood. High cholesterol and fatty diets are priming events.

The innate immune system is central to the pathology and plays a role in initiation and progression of the disease [1]. Complement (C) and complement activation are key elements of the innate immune system. There are three pathways by which the C system can be activated: classical pathway (CP), lectin pathway (LP) and the alternative pathway (AP) [2] [3]. The enzymatic generation of C3a from C3 and subsequently C5a from C5 with relevant receptors (C3aR, C5aR and C5L2) leads to complement-mediated chemoattraction of inflammatory cells. In atherosclerotic lesions, the complement anaphylatoxins C3a and C5a bind to receptors expressed by plaque intima macrophages, T cells, mast cells, endothelial and medial smooth muscle cells and therefore have a role in orchestrating the inflammatory component of atherosclerosis. Properdin is the only naturally occurring positive regulator of complement activation, leading to an amplification of ongoing complement activation. Macrophages in the atherosclerotic plaques may present as lipid laden foam cells and can be of M1 or M2 phenotype [4]. Our recent work has shown that properdin-deficient mice in comparison with their littermate wildtype controls exhibit an immune response which is compatible with a bias towards M2 activity [5].

Previous data in mouse models of atherosclerosis (ApoE−/− and/or LDLR−/− background) show the involvement of the following complement components in the pathogenesis; C3, C1q, CD59, CD55 also known as decay accelerating factor (DAF), and MBL (reviewed in [6]) with an emerging role for C3a_desArg_ in particular. C3a_desArg_, which is generated by carboxypeptidase mediated removal of the carboxyl-terminal arginine of C3a, is also known as acylation stimulating protein (ASP) due to its marked stimulating action on triacylglycerol synthesis in adipocytes.

The intact alternative pathway of complement plays a pro-atherogenic role in response to a high fat diet in mice [7]. No study has yet examined the effect of genetic deletion of properdin on atherosclerosis. Properdin is a non-enzymatic serum glycoprotein and is the only positive regulator of AP. Typically oligomers of properdin interact with Factor B bound C3b to stabilize the alternative C3 and C5 convertases that then cleave more C3 and C5.

Here, we examined the effect of genetic deletion of properdin on murine atherosclerosis in LDLR^−/−^ mice of both genders by feeding Western type diets. Our study reveals an unexpected protective effect of properdin in early atherosclerotic plaque progression.

## Materials and Methods

### Mice and Diets

LDLR^−/−^ mice (Ldlr^tm1Her^/J) on C57BL/6 background were available from Jackson Laboratories and crossbred with a previously generated properdin-deficient (P^KO^) mouse line [8]. As properdin is on the X-chromosome, the strategy was to mate female LDLR^−/−^ with male P^KO^. From their offspring, LDLR^het^ P^het^ were intercrossed with LDLR^het^ P^WT^. Their litter contained LDLR^−/−^ P^het^ and LDLR^−/−^ P^KO^, which were intercrossed in a final step to generate the experimental mice. Subsequently, breeding pairs were maintained to generate LDLR^−/−^ P^KO^ double knockouts along with LDLR^−/−^ P^WT^ mice. Polymerase chain reaction was used to confirm genotypes [8] and http://jaxmice.jax.org/strain/002077.html#genotype. All mice were used in accordance with UK legislation (1986 Animals (Scientific Procedures) Act) and were housed in a controlled environment with a 12 h light/dark cycle at 22°C. All animal experiments were carried out in strict accordance with the recommendations in the guide for the Conduct of Animal Research, National Institutes of Health. The protocols were approved by the Universities of Sheffield and Leicester Project Review Committees and carried out under UK Home Office Project Licences (40/3307).

All animals were fed a standard chow until they reached 15 weeks of age. Mice were then fed either a low-fat (LFD, Arieblok Reference Diet, cat. 4068.02, Woerden, The Netherlands, consisting of 54.3% (w/v) glucose, 10% (w/v) cornstarch, 5% (v/v) soya oil, 20% (w/v) casein, total fat content 5.2% by weight and no added cholesterol) or high-fat diet (HFD, Arieblok Diet W, cat. 4021.06, consisted of 15% (w/v) cocoa butter, 1% (v/v) corn oil, 0.25% (w/v) cholesterol, 40.5% (w/v) sucrose, 10% (w/v) cornstarch, 20% (w/v) casein, free of cholate, total fat content 16% by weight) respectively as used by others in the complement field [7]. Body weights were obtained every 4 weeks. After 12 weeks of feeding a LFD or HFD, respectively, the mice were euthanized by pharmacological overdose of pentobarbitone at the age of 27 weeks. Cardiac puncture was performed to obtain blood before hearts and aortae were perfusion-fixed with phosphate buffered saline (PBS) followed by 10% (w/v) formalin buffered saline.

### Blood Pressure Measurements

Mean blood pressure was calculated after measuring systolic and diastolic blood pressures of mice at least weekly using a Visitech tail-cuff system (Visitech Systems, NJ, USA). Weekly measurements over the entire feeding period were averaged for each individual mouse and group results were calculated as mean ± SEM.

### Atherosclerotic Plaque Assessment

Thoracic aortae were removed and fixed in 4% (w/v) paraformaldehyde (PFA, BDH) as described previously [9]. The aorta was segmented at the diaphragm and the ascending aorta. The extraneous fatty and connective tissue around the aorta were carefully trimmed off under a dissecting microscope until a clear and transparent aorta was obtained; this was then transected longitudinally. Thoracic aortae were returned to 4% (w/v) PFA for 24 h, stained with Oil Red O for 15 min and pinned onto wax. A macroscopic CCD camera was used to take images, which were analysed using computer assisted image analysis (NIS-elements/Lucia G software, Nikon UK). Total aorta and total lesion area with positive staining (mean lesion area, number of objects) were quantified for each aorta. Thereafter, area of positive staining for Oil Red O was calculated as the ratio of mean lesion area and total aorta area and expressed as percentage of total aortic area.

### Immunohistochemistry

Hearts were fixed in 10% (v/v) formalin buffered saline for at least 24 h, embedded in paraffin wax and cut serially to obtain 5–8 μm aortic root sections.

Aortic sinus sections were dewaxed in xylene and rehydrated in an ethanol series. Primary antibodies diluted as appropriate in PBS were: monoclonal mouse anti-human smooth muscle actin (SMA, Dako M0851, 1∶150, 1 h), purified anti-mouse CD206 (Biolegend 123001, rat IgG2aκ, 1∶100, 1 h RT), (trypsination for antigen retrieval: 0.1% Trypsin (DIFCO 215240) in TBS (Sigma T6664), 10 min, 37°C; 0,5% Triton-X 100) and were followed by biotinylated goat anti-mouse IgG (H+L) (Vector BA9200, 1∶500, ½–1 h) or polyclonal biotinylated rabbit anti-rat Ig (Dako E0468, 1∶500, 1 h RT) and ABC-peroxidase solution (Vektor PK6100, Vectastain Elite ABC Kit; Sigma H1009; 30 min) with 3,3′-diaminobenzidine tetrahydrochloride (DAB, Sigma D4418, 10 min) as substrate. Carazzi’s haematoxylin was used for counterstaining.

Analysis was performed using a computer assisted imaging programme (Nikon-Imaging-Software NIS-elements/Lucia G software, Nikon UK) and data were generated for mean lesion area and lesion area:cross-sectional area (CSA). Positive staining was quantified in blind fashion and expressed as percentage of total aortic area for en face Oil Red O staining and percentage of aortic CSA for SMA staining. A cell index was generated for MAC 387 and CD206 by counting positive stained cells/total cells in high power fields.

### Haematology

White blood cells (WBC), red blood cells (RBC), platelets (PLT) and cell composition of neutrophils (NEUT), monocytes (MXD) and lymphocytes (LYM) were analysed and counted in a 1∶10 dilution of whole blood in cellpack solution at 0, 6 and 12 weeks on diet using Sysmex KX-21N. Haematological parameters were expressed as concentrations, whereas cell composition was presented as percentage of cell type in whole blood.

### Lipid and Complement Biochemistry

Blood was collected via cardiac puncture into a desuridin-containing syringe (1∶100 in blood) and plasma separated by centrifugation. Triglyceride and cholesterol levels were measured at the Department of Clinical Chemistry (Royal Hallamshire Hospital, Sheffield, UK) using Roche Cobas 8000 modular analyzer series. C3 levels were measured at the Department of Infection, Immunity and Inflammation, University of Leicester, UK using ELISA specific for mouse C3 (Immune Systems Ltd, E-90C3). Briefly, C3 present in plasma was recognised by a C3-antibody which was bound to the provided microtitre wells. HRP-conjugated anti-C3 antibody, which interacts with a chromogenic substrate, was then added. C3 concentration was interpolated from a standard curve and corrected for the dilution factor (1∶50,000). The assay may also detect cleaved C3, iC3b.

Non-esterified fatty acids (NEFA) were analysed using mass spectrometry by the Department of Infection, Immunity and Biochemistry, Cardiff, UK [10]. C3adesArg was measured in a previously described sandwich ELISA using a pair of anti-mouse C3a mAb, one unlabelled as capture (0.2 μg/ml), the other biotinylated as detection (0.5 ug/ml), and recombinant mouse C3a (100 ng/ml –0.78 ng/ml) as standards (all from BD Pharmingen, San Diego, CA). This assay measures both C3a and C3_desArg_. Appropriately diluted plasma samples were included. The assay was developed with streptavidin-peroxidase (1∶5000; Jackson ImmunoResearch. West Grove, PA).

For NEFA, lipids were extracted and separated by 1-dimensional TLC on 10×10 cm silica gel G plates, double developed using toluene: hexane: formic acid (140∶60:1, (v/v)) for the entire plate followed by hexane: diethyl ether: formic acid (60∶40:1, (v/v)) to half height. Plates were sprayed with 0.05% (wt/vol) 8-anilino-4-naphthosulphonic acid in methanol and viewed under UV light to reveal lipids. Free fatty acids were scraped from the plate and identified and quantified by gas chromatography.

### Molecular Analyses on Splenocytes

For RTPCR analysis, RNA was extracted from spleens or from splenic macrophages (adherent cell population after overnight incubation of splenic single cell suspensions) and RTPCR performed. Primers used were (annealing temperatures are indicated); for C3 5′-gaatacgtgctgcccagttt-3′ and 5′-tgagtgaccaccagcacttt-3′ (55°C); for MCP-1 5′-cactcacctgctgctactcattcac-3′ and 5′-ggattcacagagagcgaaaaatgg-3′ (57°C); for sPla_2_V 5′-aagagggttgtaagtccagagg-3′ and 5′-cagggggcttgctagaactcaa-3′ (58°C); for β-actin 5′-gtgggccgctctaggcaccaa-3′ and 5′-ctctttgatgtcacgcacgatttc-3′ (55°C). Quantitative PCR for arginase (5′-gatgagaaaggaaagtggctgt-3′ and 5′-aggaactggctgaagtggttagt-3′, 215 bp, 55°C) and GAPDH (5′-cctggagaaacctgccaagtatg-3′ and 5′-agagtgggagttgctgttgaagtc-3′, 133 bp, 55°C) was performed using SensiMix SYBR kit (Bioline Reagents Ltd., London), and analysed with Rotor-Gene 6000 (Corbett Life Science). Relative expressions were calculated using the ΔΔ CT method.

### Lipid Storage Assay in Macrophages

Bone marrow derived macrophages were differentiated with 10 ng/ml rmGMCSF (Peprotech) in RPMI 1640 medium containing 10% (v/v) foetal bovine serum for seven days from cells isolated from femurs of 10–12 week old mice. They were then fixed and stained with Oil red O for 15 minutes at room temperature. Using light microscopy, cells were evaluated blinded for inclusion of Oil Red O lipid droplets.

### Statistics

All data are expressed as mean ± SEM and significance tested by two-way ANOVA and non-parametric testing with the Mann–Whitney U test (GraphPad Prism software version 5.0), with significance assumed at *p<0.05, **p<0.01, ***p<0.001, ****p<0.0001.

## Results

### Dietary Model of Mild and Advanced Atherosclerosis in LDLR^−/−^

Previous *in vivo* studies on atherosclerosis and the role of complement have used different diets and genders of mice but rarely low fat (LFD) and high fat diets (HFD) in one single study. We fed LFD and HFD to both genders of age matched LDLR^−/−^ and LDLR^−/−^ P^KO^ for 12 weeks in the same study. Body weight was increased after HFD compared to LFD but there was no difference amongst the sex-matched genotypes (Table 1). HFD led to significant hypertriglyceridemia and hypercholesterolemia, with elevated HDL and LDL cholesterol (Table 1). Female LDLR^−/−^ mice on HFD had the highest cholesterol levels, irrespective of properdin status. Levels of non-esterified fatty acids (NEFA) liberated by hydrolysis of triglycerides by hormone sensitive lipase were significantly decreased in male LDLR^−/−^ P^KO^ mice on a HFD, which had the highest mean body weight. C3 was increased in plasma of male and female LDLR^−/−^ P^KO^ versus LDLR^−/−^ P^WT^ mice fed a LFD or HFD (Table 1) consistent with impaired AP activity in LDLR^−/−^ P^KO^, which we confirmed using the standard rabbit red blood cell lysis assay (data not shown). Levels of C3a_desArg_, which are generated by cleavage of plasma C3a appeared to be lower in HFD than LFD fed male LDLR^−/−^ P^WT^ (0.57±0.10 mg/dl vs 0.76±0.05 mg/dl) but this did not reach statistical significance (Table 1). Because of the elevated C3 levels in LDLR^−/−^P^KO^, the ratio of ASP/C3 was consistently decreased by 50% in the absence of properdin regardless of diet or gender (Table 1). We also examined cell and platelet counts at six-weekly intervals (Figure S1 and S2 in File S1). Male LDLR^−/−^ and LDLR^−/−^ P^KO^ and female LDLR^−/−^ and LDLR^−/−^ P^KO^ mice had similar numbers of lymphocytes, monocytes and neutrophils at the onset of the study (LFD and HFD). The mean weekly blood pressures did not show differences between the groups after 12 weeks’ diet (Figure S3 in File S1). Fed a LFD, female LDLR^−/−^ mice at comparable cholesterol levels to male LDLR^−/−^ mice developed greater aortic lesions, as previously described [11].

**Table 1:** Body weight, complement and lipid data in male and female LDLR^−/−^P^KO^ and LDLR−/−P^WT^ fed LF and HFDs for 12 weeks.

Diet	LFD				HFD			
Gender	male		female		male		female	
genotype	LDLR^−/−^P^WT^	LDLR^−/−^P^KO^	LDLR^−/−^P^WT^	LDLR^−/−^P^KO^	LDLR^−/−^P^WT^	LDLR^−/−^P^KO^	LDLR^−/−^P^WT^	LDLR^−/−^P^KO^
**Body weight (g)**	27.4+/−0.7	28.4+/−1.1	21.5+/−0.8	21.7+/−0.5	29.9+/−1.1	31+/−1.0	23.1+/−0.5	23.0+/−0.5
**Triglycerides (mg/dl)**	111+/−11.7	105.9+/−10.2	82.9+/−11.6	84.9+/−9.6	571.7+/−64.5	573.7+/−87.2	471.4+/−35.2	412.2+/−36.9
**Cholesterol (mg/dl)**	284.4+/−26.5	272+/−11.8	270.3+/−20.3	206.8+/−9.7	1056.2+/−69.5	1067.3+/−129	1553.8+/−32.1	1713.9+/−64.9
**HDL cholesterol (mg/dl)**	95.7+/−7.6	109.4+/−6.5	84.8+/−2.9	72.3+/−5.1	139.4+/−14.2	126.5+/−8.3	246.1+/−17.4	214.5+/−20.5
**LDL cholesterol (mg/dl)**	166.6+/−22.3	141.4+/−7.7	168.9+/−18.2	117.5+/−7.6	802.4+/−62.9	826+/−109.4	1213.4+/−34.1	1416.9+/−52.3
**NEFA (mg/dl)**	15+/−2	17.3+/−1.1	17.3+/−2.1	13.5+/−2.3	35.1+/−3.7	23+/−4.6	37.2+/−1.6	31.5+/−3.8
**C3 (mg/dl)**	36.4+/−7.5	74.2+/−13.3	64.2+/−3.0	89.7+/−10.8	37.4+/−9.7	104+/−34.4	25.2+/−0.0	71.7+/−20.5
**C3desarg (mg/dl)**	0.76+/−0.05	0.81+/−0.05	0.85+/−0.08	0.81+/−0.13	0.57+/−0.1	0.56+/−0.09	0.48+/−0.23	0.84+/−0.02
**ASP/C3**	0.02	0.01	0.01	0.01	0.015	0.005	0.02	0.01

Data are means +/− SEM, n = 7–8 each group, *P<0.05.

**Figure 1:**
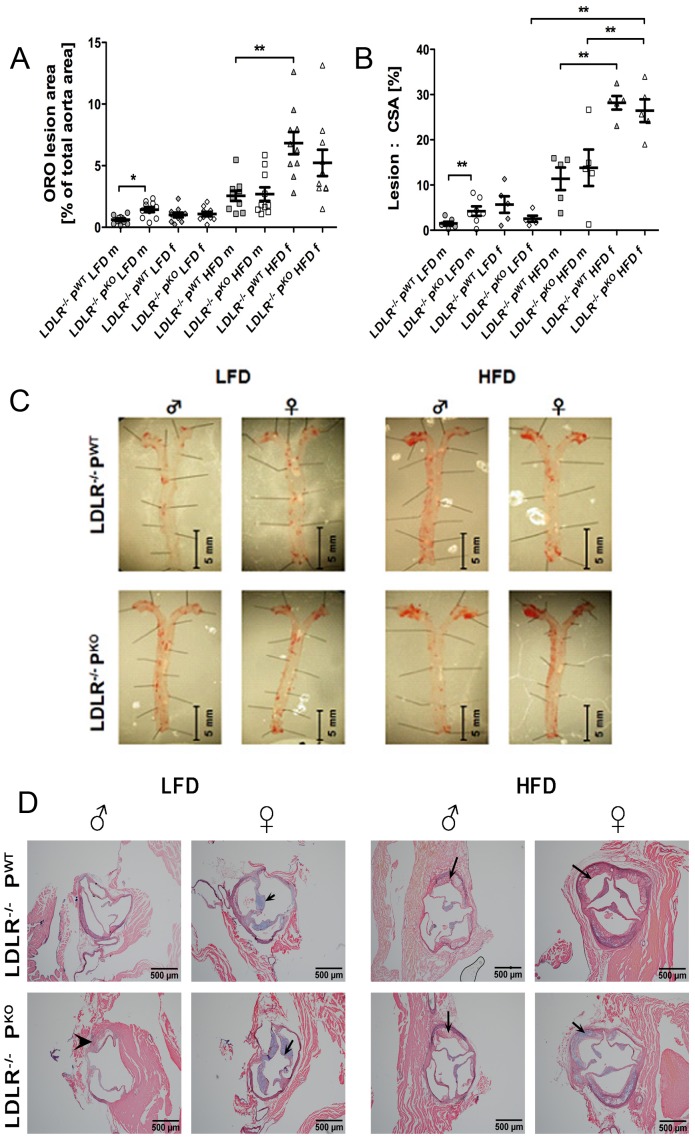
Development of atherosclerotic plaques in LDLR−/− PWT and LDLR−/−PKO mice fed LFD and HFD. **A** therosclerosis-prone mice (males and females) lacking properdin were fed low fat or high fat diets for 12 weeks. Atherosclerosis burden was assessed in aortae and at aortic roots. (**A)** lesion burden in aortae identified by Oil Red O staining (stains lipid) was calculated from light microscopic images using NIS elements software (Nikon), (8 per group, mean and IQR), *p<0.05, **p<0.01, intergenotype comparisons were by one way ANOVA with post test. (**B**) Plaque area within aortic roots measured from histological images (NIS elements), n = 5–8, data are mean and IQR **p<0.01, intergenotype comparisons were by one way ANOVA with post test. (**C**) Representative Oil Red O images of whole aortae from each group. (**D**) Visualisation of plaques at the aortic root in each of the groups, arrowhead indicates a large plaque in male LDLR^−/−^P^KO^, arrows indicate typical plaques in the other mouse groups that were studied.

We assessed atherosclerosis in aortae using an en face lipid staining method (to evaluate overall burden and distribution of disease) and at the aortic root (area of predilection for atherosclerosis in the mouse models). Lesions occurred throughout the proximal and distal aorta and the largest lesion burden in our study was in female LDLR^−/−^ P^WT^ and female LDLR^−/−^ P^KO^ mice on HFDs. This appeared to be two-fold more than their male counterparts on HFD (Fig. 1A). The en face oil red O staining for lesion burden within whole aortae showed that properdin was protective only in male mice fed a LFD (Figure 1A). This protective effect was overwhelmed by feeding of a HFD (Figure 1A). Atherosclerotic plaque formation at the aortic root was also significantly larger in male LDLR^−/−^P^KO^ mice fed a LFD compared with wild type littermate controls (Figure 1B). Plaques were distributed along the thoracic aorta (Figure 1C) and plaque formation was within subendothelial layers (Figure 1 D).

### Properdin Dampens Development of Atherosclerosis in Male LFD fed LDLR^−/−^

**Figure 2:**
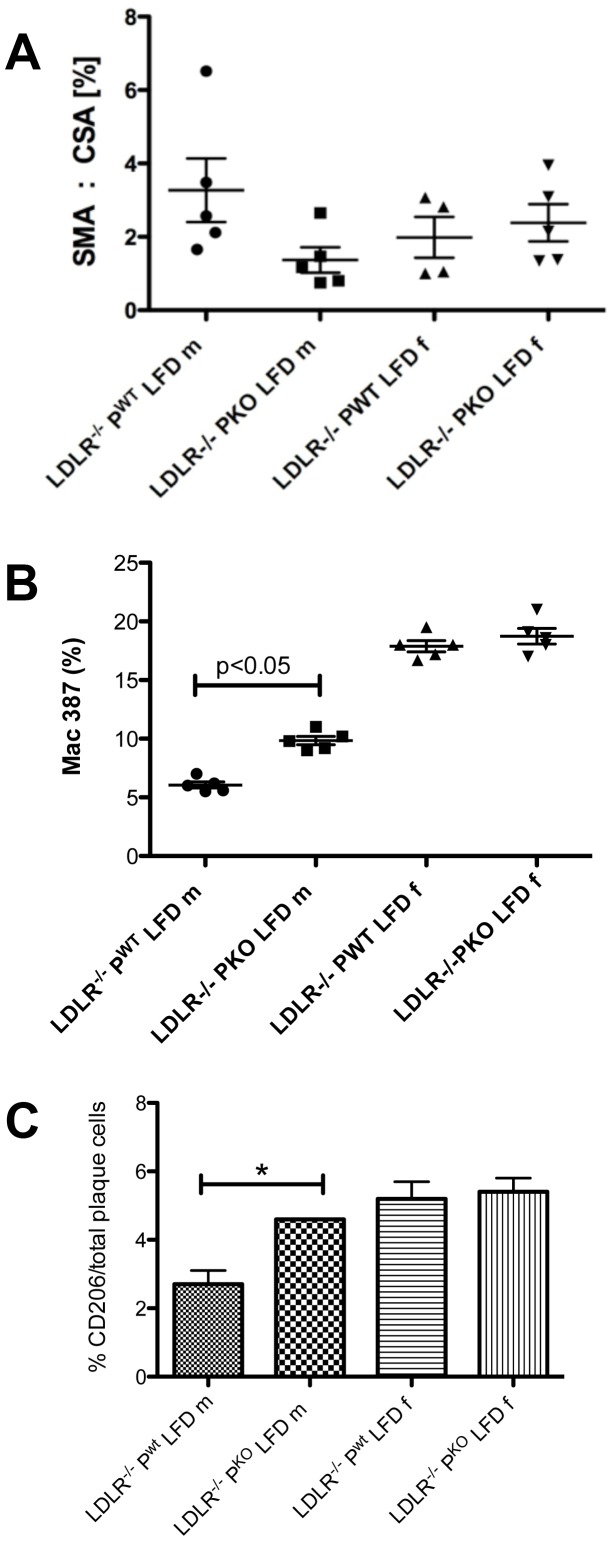
Properdin deficiency promotes increased macrophages in atherosclerotic lesions after feeding on a low fat diet. Quantitative immunohistochemistry of (**A**) % smooth muscle cells in aortic root plaques of male and female LDLR^−/−^P^KO^ versus LDLR^−/−^P^WT^ mice. Random areas in 15–20 plaques from n = 4–5 mice/group were analysed under high magnification using NIS elements software. Data presented are mean and IQR (**B**) Macrophage (MAC 387) index (percentage of the total cells/high power field within plaques) from the relevant groups, n = 5 mice/group were examined, mean and IQR, p<0.05, intergenotype comparisons were by two way ANOVA with a Mann Whitney U post test. (**C**) M2 (CD206) macrophage index, n = 5 mice/group examined, mean +/− SEM, p<0.05 (2 way ANOVA and Mann Whitney U test).

Subsequent analyses focussed on LDLR^−/−^P^KO^ and LDLR^−/−^P^WT^ mice fed LFD since the largest effects of the properdin genotype were seen in mild rather than advanced atherosclerosis. Immunostaining of aortic sinus plaques from male and female LDLR^−/−^P^KO^ and LDLR^−/−^P^WT^ mice for smooth muscle cell actin (smooth muscle cells), high resolution imaging and subsequent analysis showed that there were no significant differences in the % VSMC/plaque area between these groups (Figure 2A) although a trend for lower SMA in LDLR^−/−^P^KO^ compared with LDLR^−/−^P^WT^ mice was observed. In contrast, there was a significant increase in the % macrophages/plaque area between male LDLR^−/−^P^KO^ and LDLR^−/−^P^WT^ (Figure 2B). Further, CD206 (mannose receptor) staining revealed a significant increase in M2 macrophages within LDLR^−/−^P^KO^ plaques in male mice fed a LFD compared with LDLR^−/−^P^WT^ littermates (Figure 2C). This difference was not seen in female mice fed a LFD.

The greater plaque area in LDLR^−/−^ P^KO^ mice appears not to be connected with differences in plasma cholesterol or triglycerides since these were similar amongst groups (Table 1). However, we noted that at the endpoint of the experiment, male LDLR^−/−^ P^KO^ mice had significantly elevated platelets and depressed neutrophils compared to LDLR^−/−^ P^WT^ mice (Figure S2C and Figure S1C in File S1).

**Figure 3:**
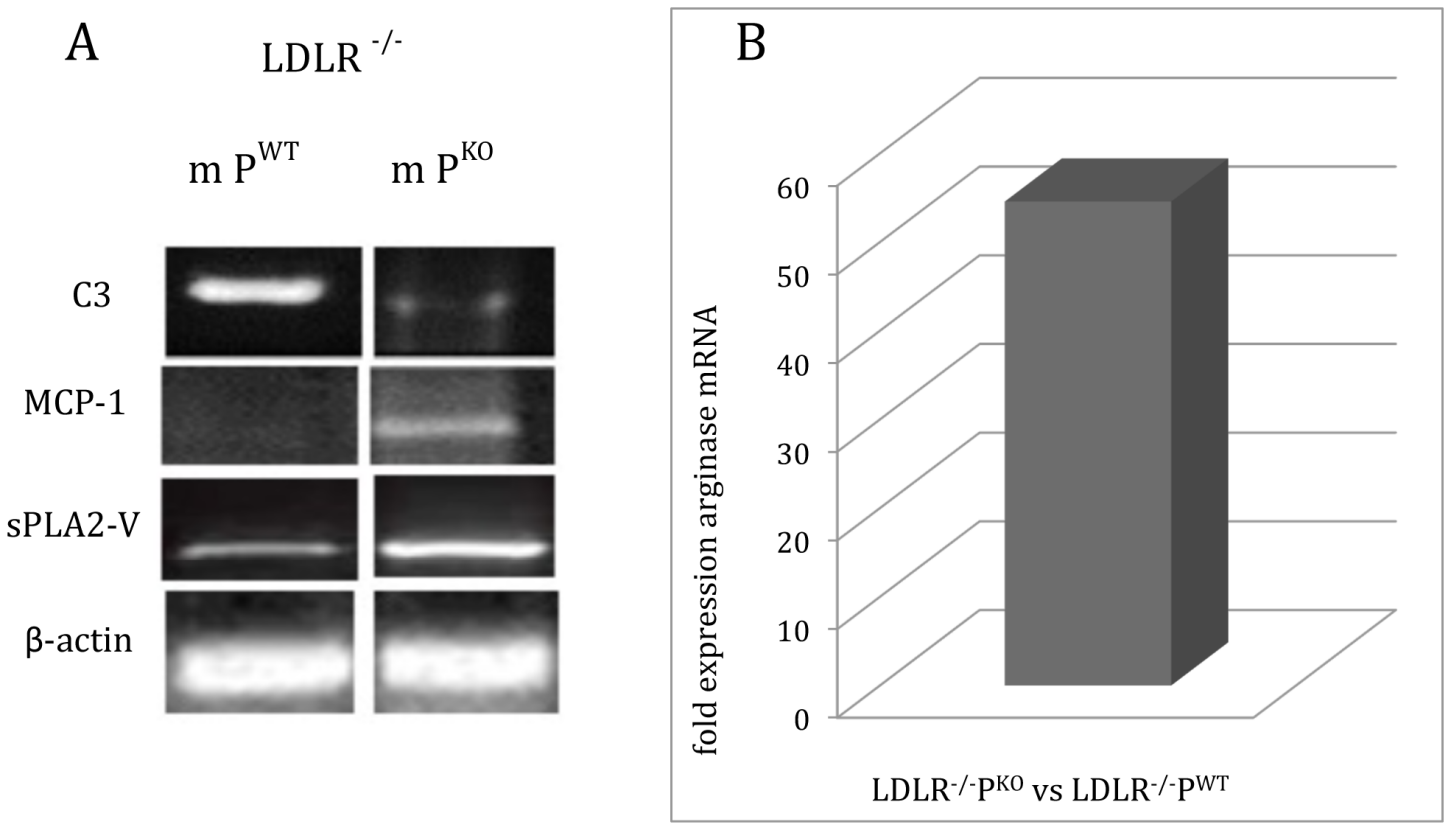
Properdin deficiency promotes macrophage mRNA synthesis typical of M2 macrophages under conditions of atherosclerosis. (**A**) Spleens from male LDLR^−/−^P^KO^ and LDLR^−/−^P^WT^ fed a LFD were analysed for various mRNA species including C3 (500 bp), sPLA2-V (329 bp) and MCP-1 (490 bp) by RTPCR. β-actin (540 bp) was control. (**B**) Arginase 1 mRNA expression was enhanced 50 fold in splenic macrophages from male LDLR^−/−^P^KO^ compared to LDLR−/−P^WT^ fed a LFD. Representative images and analyses of matched pairs are shown.

Because macrophage numbers, especially M2 macrophages were increased in lesions (Figures 2B and 2C), we also examined macrophage function. The ability of bone marrow-derived macrophages from male LDLR^−/−^P^KO^ and LDLR^−/−^P^WT^ to store lipid droplets was examined *in vitro*. LDLR^−/−^ P^KO^ macrophages had nearly twice the storage capacity of LDLR^−/−^P^WT^ macrophages (Table 2). To investigate systemic correlates of greater accumulation of macrophages in LDLR^−/−^P^KO^ atherosclerotic lesions (LFD group), the expression of a number of signature genes for M2 macrophage polarisation was examined. MCP-1 and sPLA2-V mRNA expression were increased in male LDLR^−/−^P^KO^ fed a LFD compared with LDLR^−/−^P^WT^. C3 mRNA expression, consistent with the systematic analysis conducted by others for M1 and M2 macrophages in parallel [12], was expectedly decreased in macrophages from LDLR^−/−^P^KO^ (Fig. 3A). Further, arginase 1, a marker of alternatively activated (M2) macrophages in experimental models of atherosclerosis, was increased 50 fold at the mRNA level in splenocytes of LDLR^−/−^P^KO^ compared with LDLR^−/−^P^WT^ (Fig. 3B). Taken together, properdin deficiency on LDLR^−/−^ background is associated with increased expression of M2-type macrophage inflammatory response in aortic lesions and in the systemic compartment.

**Table 2:** Enhanced lipid storage in properdin-deficient macrophages isolated from pro-atherosclerotic mice.

	LDLR^−/−^P^WT^ 648 cells counted	LDLR^−/−^P^KO^ 610 cells counted
Oil Red O pos cytoplasm	18.2%	32.6%
Oil Red O neg cytoplasm	81.8%	67.4%

Bone marrow derived macrophages (BMDM) from male LDLR^−/−^P^KO^ mice have the capacity to store more LDL as lipid droplets within cells than LDLR^−/−^P^WT^. 610–648 cells counted.

## Discussion

Previous *in vivo* studies have concluded that complement activation plays a role in atherosclerotic lesions. However, the overall role of complement is complex and the positive regulator of C3 activation, properdin, has not been studied in experimental atherosclerosis. Our comprehensive study indicates a protective effect of properdin in male mice but only under conditions of ‘mild’ atherosclerosis. We show that as a result of properdin deletion on a hyperlipidemic background, circulating complement C3 accumulates, alternatively activated (M2) macrophage infiltration in aortic lesions is enhanced and lesion size is increased. In more advanced disease with significant hypercholesterolemia, this protective effect is overwhelmed by other systems and signalling pathways.

C3 behaves as an acute phase reactant similar to CRP and is increased in atherosclerotic lesions [13]. C3 deficient mice have significantly larger atherosclerotic lesions than LDLR^−/−^ C3 sufficient controls [14], [15] but not Factor B deficient mice on ApoE^−/−^ LDLR^−/−^ background [14]. This concurs with Malik *et al*. [7], who studied Bf^−/−/^LDLR^−/−^ and found no difference in serum C3 levels and no difference in atherosclerotic lesion development compared to LFD fed Factor B sufficient controls. However, on HFD, Bf^−/−/^LDLR^−/−^ developed elevated C3 levels but fared better than WT, possibly due to a reduction in cholesterol.

There are methodological differences between the study by Persson [14] and our study. For example, Persson *et al.* did not use fat feeding but rather double transgenics LDLR^−/−^ ApoE^−/−^ spontaneously developing atherosclerosis on a mixed C57BL/6/129Ola background. The methodology used by Persson also contrasts to the study by Malik [7] who studied female mice on LDLR^−/−^ background (strain C57BL/6) and fed LF and LF with LPS and LPS plus HF diets. Our comprehensive study reported herein used the LDLR^−/−^ background, male and female mice and low and high fat diets. We used both genders since gender specific differences in complement studies in mice are well recognised [16] and complement proteins appear to be variably influenced by gender and different decay rates of C3b/iC3b have been reported between female and male mice [17]. Our study is unique in that both genders of mice on two different diets have been studied.

Complement proteins might reasonably be expected to alter lipid levels in pathological situations [6]. In our study, there was no impact of properdin upon these. Interestingly, LDLR^−/−^ P^KO^ male mice had reduced NEFA levels compared to their corresponding wild type mice on HFD, but the reduction did not impact favourably on the size of aortic lesions. Elevated NEFA have previously been shown to modulate atherosclerosis in a mouse model although the exact mechanisms involved are unclear [18]. Although we detected an altered function of LDLR^−/−^ P^KO^ BMDMs to store more lipid in an *in vitro* assay, we saw no increases in the size of the lipid cores in lesions in our model. Lesional macrophages and in particular CD206^+^ macrophages, were increased in male LDLR^−/−^ P^KO^ mice. While the basal mRNA expression for C3 was lower in macrophages from LDLR^−/−^ P^KO^ mice than in those from LDLR^−/−^ P^WT^ mice, additional sources of C3 production (adipocytes, hepatocytes) in combination with reduced C3 conversion in the absence of properdin are likely to account for the elevated C3 protein levels we measured in LDLR^−/−^P^KO^ versus LDLR^−/−^ P^WT^ mice.

Contrasting with the increase in C3 in LDLR^−/−^P^KO^ versus LDLR^−/−^ P^WT^ mice, there was no difference in its cleavage product C3a_desArg_, also known as ASP. Since there has been increasing interest in C3 cleavage products such as C3a_desarg_ (ASP), which has been linked to obesity, we measured weight gain in our study. Although female mice had more atherosclerosis, male mice had a consistently higher body weight than females in this study but deletion of properdin had no overall significant effect upon this or the weight of fat pads in either gender (data not shown). At the mid-point of the experiment, atherosclerotic male LDLR^−/−^ P^KO^ mice showed a trend towards less weight gain. This tentatively suggests that properdin might have transient effects on metabolism but that any effects were subtle on this atherosclerotic background.

ASP is produced from C3 by adipsin and Factor B. Properdin stabilizes the C3 convertase [19] so in the presence of properdin there should be more ASP and less in its absence. Our data suggest that in all diets and genders of mice, in the absence of properdin, there is a decrease of up to 50% in the ratio of ASP/C3, consistent with less production of ASP. Properdin was without direct effect on ASP possibly because of variance in the fat composition in the diet compared to other studies [20] and/or the alterations in mechanisms of clearance of lipoproteins due to the absence of LDLR. ASP is considered to be critical for triglyceride synthesis and the maintenance of metabolic homeostasis [21], [22], [23] although the importance of C3a_desArg_ in systemic lipid metabolism remains controversial and has been contradicted in previous studies [15].

Hematological parameters were not changed throughout the course of this study. While circulating factor D (of AP) inhibits thrombin-induced platelet aggregation in vitro [24] there was no effect of properdin deficiency on platelet number or activation at any time point.

In terms of atherosclerotic plaque burden, properdin was protective in male mice but only when fed a LFD. This concurs with the study by Persson [14] who also found that positive effects of C3 deletion were overwhelmed at later time points, 26 weeks in that specific study. The authors linked the early phenotype to the possible presence of neutrophils in lesions and since properdin is produced by neutrophils [25] this could also play a role in our study.

This early effect of properdin on lesions points to a potential role in ‘mild’ rather than ‘moderate’ or ‘complex’ lesion formation. In our study, there was no increase in VSMC whose excessive proliferation as part of the stereotypic atherosclerotic process causes thickened vessel walls and accumulation in plaques [26]. Only the % of macrophages in lesions and their phenotype appeared altered by properdin deletion, with enhanced lipid storage function and increased expression of CD206, MCP-1 and arginase, supporting a likely M2 phenotype [27]. M2 macrophages account for approximately 20% of the total macrophages in lesions from LDLR−/− mice fed a Western diet for 30 weeks [28] but implications of this are not yet widely understood. It is known that M2 macrophages are involved in tissue remodelling, repair and the resolution of inflammation [29] and that macrophage switching in lesions is known to be an early phenomenon in the life cycle of plaque development in mouse models [30]. We suggest that the balance of M1/M2 vs other M types and their individual functions and interactions with other cell types e.g. neutrophils, ultimately could determine overall lesion size and dynamics.

Interestingly, the development of the M2 macrophage phenotype is under the control of estrogen and progesterone [31], which could explain the greater abundance of lesional CD206^+^ cells observed for female LDLR^−/−^ in our study. The percentage of M2 macrophages in aortic root plaques was, however, not increased further in the absence of properdin (fig. 2C). In keeping with suggestions from Bacci and Fujisaka et al. [32], [33] that M2 macrophages are over recruited under conditions of a high fat diet especially when metabolic disturbance e.g. insulin resistance and weight gain occurs, we find the greatest Oil red O lesion areas, indicative of foam cells, in HFD fed female and male mice (Fig. 1A).

In conclusion, this definitive study indicates that the positive regulator of the alternative complement pathway has anti-atherogenic effects in an unstressed (mild lesion) situation. This effect appears restricted to male mice only and is overwhelmed by feeding a HFD. We postulate that normal levels of C3 and the presence of properdin appear to be protective in experimental atherosclerosis. Properdin has a unique role in the complement system and further studies directed at more metabolic aspects of atherosclerosis may be valuable.

## Supporting Information

File S1 **Figure S1. Cell composition: Lymphocytes (LYM, A), monocytes (MXD, B) and neutrophils (NEUT, C) in whole blood of diet-induced atherosclerotic LDLR^−/−^ P^WT^ and LDLR^−/−^ P^KO^ mice on LFD and HFD.** Data from 0, 6 and 12 weeks on diet and each set of 3 bars represents these times. Bars represent Mean ± SEM, numbers below bars are the number of mice. Statistical analysis was performed with two-way ANOVA. *P<0.05. **Figure S2.** **Haematological parameters: white blood cells (WBC, A), red blood cells (RBC, B) and platelets (C) in whole blood of diet-induced atherosclerotic LDLR^−/−^ P^WT^ and LDLR^−/−^ P^KO^ mice on LFD and HFDs.** Data have been collected at 0, 6 and 12 weeks on diet and each set of 3 bars represents these times. There are some decreases in platelet number between P^WT^ and P^KO^ on LFD but these resolve over time up to 12 weeks. Bars represent Mean ± SEM, numbers below bars represent number of mice. Statistical analysis was performed with two-way ANOVA. *P<0.05, **P<0.01, ***P<0.005. **Figure S3. Blood pressure measured using tail cuff plethysmography.** **A**) male mice, **B**) female mice, fed a low and high fat diet with or without Properdin deletion. Data represent Mean ± SEM. There were no statistical differences using two-way ANOVA.
(DOCX)
