# Supplementary figures and images for: Protective Role for Properdin in Progression of Experimental Murine Atherosclerosis
Source: PLoS One. 2014 Mar 25;9(3):e92404. doi: 10.1371/journal.pone.0092404 (PMC3965423; doi:10.1371/journal.pone.0092404)

Figure S1


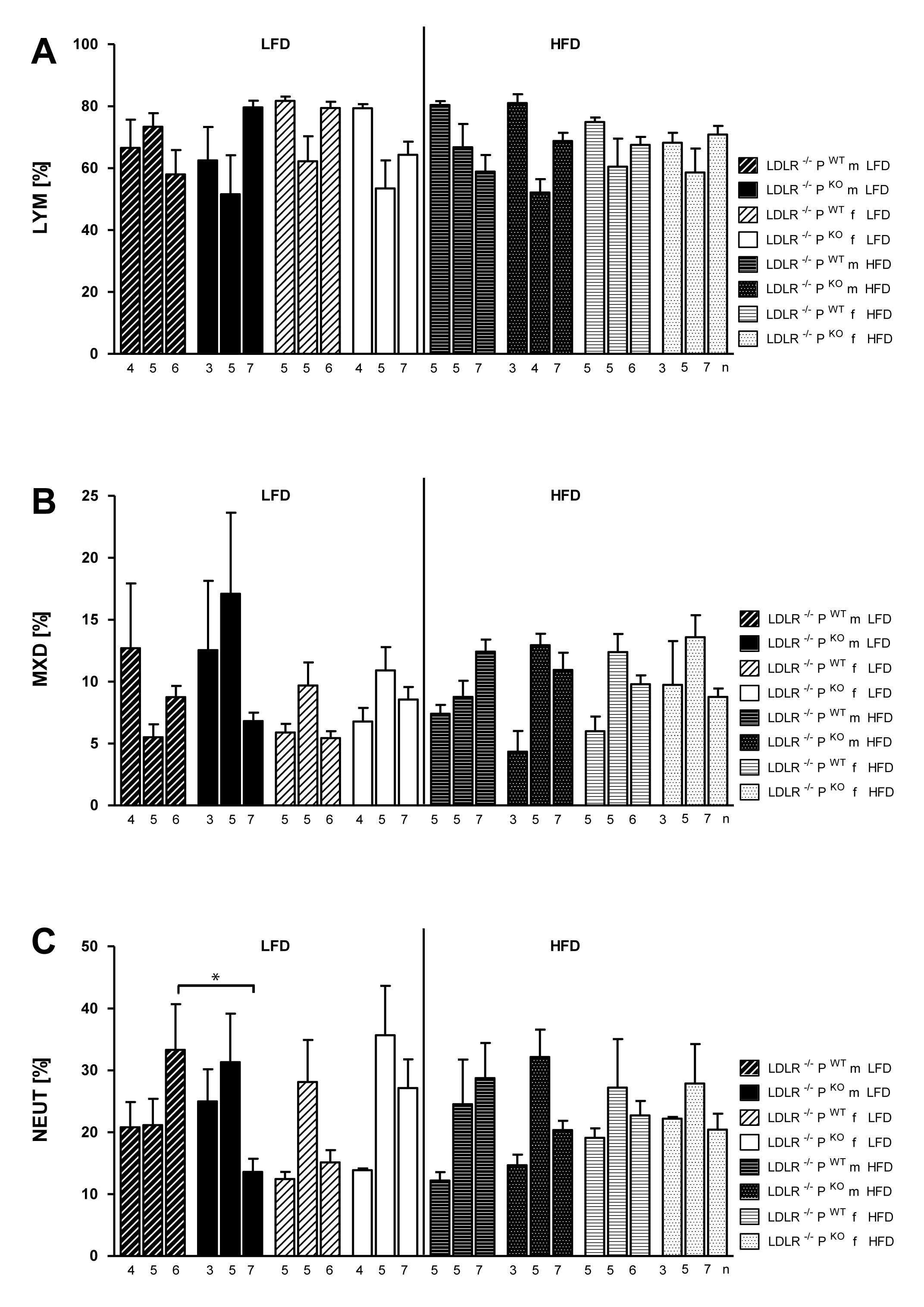


Figure S2

Figure S3

Supplement: File S1 — Figure S1. Cell composition: Lymphocytes (LYM, A), monocytes (MXD, B) and neutrophils (NEUT, C) in whole blood of diet-induced atherosclerotic LDLR−/− PWT and LDLR−/− PKO mice on LFD and HFD. Data from 0, 6 and 12 weeks on diet and each set of 3 bars represents these times. Bars represent Mean ± SEM, numbers below bars are the number of mice. Statistical analysis was performed with two-way ANOVA. *P<0.05. Figure S2. Haematological parameters: white blood cells (WBC, A), red blood cells (RBC, B) and platelets (C) in whole blood of diet-induced atherosclerotic LDLR−/− PWT and LDLR−/− PKO mice on LFD and HFDs. Data have been collected at 0, 6 and 12 weeks on diet and each set of 3 bars represents these times. There are some decreases in platelet number between PWT and PKO on LFD but these resolve over time up to 12 weeks. Bars represent Mean ± SEM, numbers below bars represent number of mice. Statistical analysis was performed with two-way ANOVA. *P<0.05, **P<0.01, ***P<0.005. Figure S3. Blood pressure measured using tail cuff plethysmography. A) male mice, B) female mice, fed a low and high fat diet with or without Properdin deletion. Data represent Mean ± SEM. There were no statistical differences using two-way ANOVA. (DOCX) [file pone.0092404.s001.docx]
